# Variation in Health Status Reports: Triangulating Mixed Methods Data to Assess the Health and Wellbeing of Primary Caregivers to Older Rural South Africans

**DOI:** 10.1007/s10823-025-09545-w

**Published:** 2025-10-16

**Authors:** Michelle Brear, Themby Nkovana, Guy Harling, Lenore Manderson

**Affiliations:** 1 MRC/Wits Rural Public Health and Health Transitions Research Unit, School of Public Health, University of the Witwatersrand, Johannesburg, South Africa; 2 Monash University, Melbourne, Australia; 3 University College London, London, United Kingdom; 4 MRC/Wits Rural Public Health and Health Transitions Research Unit, School of Public Health, University of the Witwatersrand, Johannesburg, South Africa; 5 School of Public Health, University of the Witwatersrand, Johannesburg, South Africa

**Keywords:** Self-rated health, Caregiving, Mixed methods, Quality of care, Disparities

## Abstract

Caregivers’ health status is important, given its importance for their own wellbeing and capacity to provide quality care. While single item self-rated health questions in surveys are an efficient measure, responses limit understanding of what people mean when they rate their health in a particular way, and do not address reporting heterogeneity. We draw on data collected in a mixed-method study on the informal caregiving of older people in rural northeast South Africa, which including a standard cross-sectional quantitative survey, an ethnographic survey, and longitudinal ethnographic observations. Results indicate that who becomes the caregiver, and the form of care provided, are influenced primarily by conventional expectations of gender, age, and kinship, and of caregiving alternatives. Caregivers invoke the social circumstances in which they provide care when describing and rating their own health and ability to care, and in determining what conditions they include or dismiss as indicators of health or illness. Social context influences respondents’ evaluation of own health and capacity to care, future ability and needs, including as reported in response to different methods. We advocate carefully constructing health condition response categories to include functional impairments and to be informed by context.

**What this pape** **r adds:**

- Contrasts three different methods to illustrate how time and social relations shape results
- Illustrates the importance of context for respondents’ evaluation of own health and capacity to care
- Highlights how conventional expectations of gender and kin, and caregiving alternatives, influence who becomes the caregiver and the form of care provided.

**Applications:**

- Survey questions about health status can be strengthened by including conditions and/or symptoms informed by ethnographic research, such as reduced hearing or vision, and mobility and dental problems, which may not otherwise be reported by respondents
- Enquiring about these functional changes may identify difficulties in caregiving and reduced capability of the caregiver, allowing for timely interventions by local services.
- There is potential value in building healthcare structures that reflect community patterns of interaction, including home visiting or community-led monitoring of at-risk homes or individuals, so to increase the capacity of individuals to care well for frail older householders.

Caregivers’ health status is important for their own wellbeing and because it can influence their capacity to care for others. Efficient measures that produce valid and reliable quantitative data about caregiver health status in LMIC contexts are needed to inform policies and programs (Dobreva & Posel, 2023). However, people affected by disease who might objectively be considered in poor health, when asked, often rate themselves healthy (Balaj & Eikemo, 2022; Mojola et al., 2022). Caregivers especially provide positive health status assessments compared to ‘objective’ behavior (e.g. drug intake), disease diagnosis, or symptom-based measures of health (e.g pain), possibly because they reference their own health to that of their care recipient (Bom et al., 2019). Political and economic factors beyond the health system, such as social welfare or employment opportunities, also influence the ways in which people rate their own health (Balaj & Eikemo, 2022), as well as capacity to maintain good health status (Medvedyuk & Raphael, 2023).

Screening for or asking people to report chronic and other health conditions with which they have been diagnosed is commonly used as an ‘objective’ measure of health in surveys (World Health Organization [WHO], 2018). Multi-item, self-rated health measures correlate better with measures of chronic disease and population-level life expectancy compared to single item measures (van Ginneken & Groenewold, 2012). Yet single item self-rated health (SRH) measures are efficient (Dobreva & Posel, 2023), and although subjective, are strong predictors of mortality (Jylhä, 2009). The single item SRH is commonly used in health-related survey research, with participants asked to identify on a simple 5-point Likert scale: “How would you rate your health today: very good, good, moderate, bad or very bad?” Single-item SRH questions provide a snapshot of health for, and as conceptualized by, an individual. Yet perceptions of own health, like health itself, are products of social circumstance (Balaj & Eikemo, 2022; Medvedyuk & Raphael, 2023). Individuals with the same objective health status (e.g. same number of chronic health conditions), who differ in terms of cultural background, social circumstance, health-related behavior and/or educational attainment, may rate their health differently in response to single item SRH measures (Balaj & Eikemo, 2022; Jylhä, 2009).

An emerging literature is concerned with self-ratings of health, with the aim to improve reliability, validity and interpretations of this common and easy to use health measure (Dobreva & Posel, 2023). Unemployment, dependence on social grants, poverty, household structure, and access to services and infrastructure, may all affect self-ratings (Balaj & Eikemo, 2022; Ice et al., 2022). Inconsistency in self-reports compared with objective measurements, and according to gender, class, race, and country, have been reported for people experiencing a range of diseases and functional impairments (Calvey et al., 2024; Lewis et al., 2025; Li et al., 2024; Xiao et al., 2024). In a nationally representative South African sample, there was heterogeneity in self-reports by gender, with women consistently providing lower SRH scores compared to men, despite mortality being higher among men (Dobreva & Posel, 2023). Similarly, among Black rural South Africans, although there was no significant association between SRH rating and disease status (Mojola et al., 2022), gender was significant. Men use negative health narratives to make sense of their “troubled livelihoods” and structurally determined inability to achieve traditional – breadwinner – masculinities; some women (lacking financial and social support) use negative health narratives to explain difficulties performing gendered domestic work (Ice et al., 2022). In contrast, in three socio-culturally diverse European countries, class seemed especially influential: people of lower socio-economic status consistently rated their health as worse than those of higher socio-economic status with the same number of health conditions (Balaj & Eikemo, 2022).

These discrepancies point to a need to better understand what informs people’s self-ratings of health. This includes for people whose activities depend on them being ‘well enough’ to undertake particular roles, in this case, caregiver roles. Our aim in this article is to enhance understanding of the meanings South African caregivers of older people attributed to health, when asked to rate their own health in a survey. Drawing on data from a mixed methods study in rural South Africa, our objectives are to:

- Compare caregiver health observed in an ethnographic study with health status reported through a standard survey and an ethnographic survey.
- Examine ethnographic case studies to better understand what caregivers meant when they rated themselves as healthy in the standard survey.

## Study Design

### Setting and Sample

The study on which we draw was conducted in northeastern Mpumalanga, South Africa. With population ageing, increasing numbers of older people who are frail or have functional impairment and cognitive decline require home-based care. This includes people living with Alzheimer’s disease and related dementias (ADRD). HAALSA (Health and Ageing in Africa—a Longitudinal Study in South Africa), until 2023 referred to as HAALSI, is a population-based cohort which has since 2014 included a sub-study of the prevalence and incidence of cognitive decline, known as HAALSA-Dementia). Using data from HAALSA-Dementia, we sampled the nominated primary caregivers of 116 people predicted algorithmically to have mild cognitive decline or moderate-severe dementia (designated the care recipient) (Manderson et al., 2022). The unemployment rate is high among all residents, and many depend on government child support, disability and old age social grants, and remittances from family members who have moved away for work (Ginsburg et al., 2021; Ralston et al., 2015).

Our mixed methods study included two concurrent components, a quantitative survey and an ethnography (Manderson et al., 2022). Each person was invited to complete a standardized survey to elicit caregiver demographic, health, caregiving and social support information, and willingness to be invited to participate in the ethnographic study. The survey was translated from English to Xitsonga by a person familiar with both formal health terminology and Xitsonga equivalents, administered to the primary and other caregivers of each care recipient, by a team of four Xitsonga-speaking fieldworkers over a six-month period (July-December 2022). Our ongoing analysis focuses on understanding associations between different topics of enquiry, including how caregiving influences the health of caregivers and the mediating role of social networks in their own health.

Alongside this survey, 21 of the 116 primary caregivers were recruited to participate in an ethnographic study. Purposive sampling was used to maximize diversity in age, gender, relationship to care recipient and severity of care recipient’s cognitive decline, as predicted by their HAALSA-dementia score. Male and non-familial paid primary caregivers are not common, and they were intentionally over-represented to ensure they were included in the ethnographic sample.

### Data Collection and Datasets

#### Multiple Methods Project and Mixed-method Data Set

The mixed-method dataset analyzed below derives from the standard quantitative survey, and from ethnographic methods (participant observation, audio-recorded semi-structured interviews, a ethnographic survey (described further below) and informal conversations with caregivers and others in the field setting). The standard survey was designed to generate knowledge about caregiving, social networks, and care recipient health. We also collected caregiver health, social and economic data that might impact their capacity to care or point to difficulties in association with it, such as total number of household residents, sources of income, reported anxiety and depression, and social isolation. The survey instrument included standardized demographic, caregiving, social network and health modules. Ethnographic research generated rich data about the context and complexity of caregiving.

#### Core Qualitative Dataset

The qualitative component included transcribed fieldnotes of > 300 h of interactions, including multiple informal interviews, with all 21 primary caregivers. We audio-recorded formal interviews with only nine primary caregivers, as we observed caregivers becoming overly formal, withholding information and “performing” when we turned on the audio recorder; they did not do this when we interviewed them informally and documented the interviews using field notes. These data were collected by Brear and Nkovana, who visited all participants in their homes frequently for 9–12 months (August 2022 to July 2023) during and following the quantitative survey data collection. Brear and Nkovana also accompanied the caregivers and their care recipients on outings, especially to health facilities but also to other government offices, to visit relatives, and to events such as funerals.

#### Supplementary Quantitative Data

The standard survey included several self-rated health questions, some stand-alone and others health-specific, drawn from multi-item scales intended to measure caregiver burden and related constructs (Table 1). Responses to the selected questions provide an overview of each caregiver’s self-rated physical and psychological health on the day of, and immediately prior to, completing the survey. An ethnographic survey was designed to enquire about issues identified as salient for one or more caregivers participating in the ethnography and implemented in June-July 2023. In this survey, caregivers were asked about any chronic health conditions or problems they experienced, in contrast to the standard survey which had generated data about conditions identified by a healthcare worker. We also enquired about conditions or problems not included in the standard survey, but reported by the ethnography participants, including arthritis, pain, menopausal hot flushes, dental problems, and limitations in vision, mobility, and hearing.

**Table 1:** Kaya questions relating to self-rated health

Question	Response options	Source
I am healthy enough to care for CR	• Strongly disagree • Disagree • Neither agree nor disagree • Agree • Strongly agree	Single item within Caregiver Reaction Assessment scale
My health has gotten worse since I've been caring for CR	• Strongly disagree • Disagree • Neither agree nor disagree • Agree • Strongly agree	Single item within Caregiver Reaction Assessment scale
In general, how would you rate your health today?	• Very good • Good • Moderate • Bad • Very bad	Stand-alone
How often do you feel your health has suffered because of your involvement with CR?	• Never • Rarely • Sometimes • Quite frequently • Nearly always	Single item within Zarit Burden scale
Have you ever been told by a doctor, nurse, or other healthcare worker that you have any of the following?	• HIV • High blood pressure or hypertension • Raised blood sugar or diabetes • Stroke • Angina (chest pain due to heart disease) • Heart attack • High cholesterol • Kidney disease • Cancer • COPD or asthma • Alzheimer’s disease or memory problems • Parkinson’s disease	Stand-alone
Do you have any other health problems?	• Free text	Stand-alone

Among the 21 caregivers, two thirds (14, 67%) completed the survey; seven did not because they had moved from the study community (3), were still living locally but had started working fulltime (2) or were ill (2) in the final month of the ethnography, when the survey was implemented. There is no way to assess what may have been different between caregivers who did and did not complete the ethnographic survey, yet no reason to think that the differences would have been systematic. Longitudinal observations showed that many of the seven caregivers who did not complete the ethnographic survey experienced health problems such as pain that were not measured in the standard survey.

### Data Analysis

#### Analysis of Quantitative Data

We interrogated the quantitative data of responses provided by the 21 index caregivers who participated in the ethnography with their responses to select questions from the standardized survey (listed in Table 1) and, for 14 of these caregivers, to the health questions in the ethnographic survey. This approach, referred to as “crossover qualitative analysis” (Onwuegbuzie & Combs, 2010), involved examining each participant’s responses and identifying inconsistencies across the data collection methods.

#### Analysis of Qualitative Data

We analysed the qualitative data over time to explore the complexity of caregivers’ health, drawing on our understanding of circumstances derived from the ethnographic research. The caregiver health dataset and general qualitative data were analyzed on a case-by-case basis and compared with the quantitative data for each case. Three inter-related and established techniques for enhancing rigor in qualitative research—prolonged engagement, persistent observation and production of thick, rich data – were embedded into the study design. We did not use multiple coders or attempt to achieve inter-rater reliability, as these techniques are intended to enhance rigor in studies that use semi-structured interviews for data collection (Morse, 2015). Quantitative and qualitative results were integrated as appropriate to assist with interpretation. Cases were then compared and the data interrogated for insights regarding the meanings caregivers attributed to “health” and related terms. Recurring patterns, as well as infrequent but salient ideas, were identified. The results were interpreted with a view to understanding the meanings caregivers attached to questions about health. We then selected illustrative cases, described below and identified by pseudonym to protect confidentiality. Qualitative cases were selected because they represented illustrations of various reasons (downplaying health problems so as not to alarm the care recipient, changes in health status, etc.) for discrepancies between self-rated and “objective” or observed health status, and/or capacity to care.

Throughout the study we applied ethical concepts such as autonomy and beneficence in practice, for example by viewing consent as an ongoing process, rather than a once-off form-signing procedure and constantly reflecting on the ethical dimensions of our interactions with participants (Guillemin & Gillam, 2004). This study was approved by the Human Research Ethics Committees of the University of the Witwatersrand, Mpumalanga Province Health Department – Health Research Committee, and University College London.

## Findings

### Caregiver Demographics and Care Recipients

The 21 primary caregivers involved in the ethnography were aged 21 to 90 years old; three were male, 18 female. Two were non-familial paid caregivers; 19 were related to the care recipients as grandchildren (6), children or children-in-law (5), wives (3), sisters (2) and other relatives (3). Care recipients ranged in age from 65–94, with roughly half each male and female. They experienced a range of health conditions, but only two were reported by their caregivers or observed by Brear and Nkovana to have psychological or behavioral symptoms suggesting cognitive impairment. This is, as indicated above, despite that all caregivers had been recruited because the care recipients had been identified as at risk of or living with mild-moderate dementia (Table 2).

**Table 2:** Pseudonyms and characteristics of caregivers in the ethnographic sample

Pseudonym	Age (years)	Gender	Relationship to CR	Standard survey responses		Combined health problem count *
				Self-rated health	Healthy enough to care for CR?	
Khensani	30	F	Granddaughter	Very good	Strongly agree	0
Hayley	26	F	Granddaughter	Very good	Somewhat agree	2
Masana	24	F	Granddaughter	Moderate	Strongly agree	3
Isaac	37	M	Son	Very good	Somewhat agree	2
Lulama	57	F	Paid caregiver	Good	Somewhat agree	4
Luleka	29	M	Grand nephew	Very good	Strongly agree	1
Saseka	28	F	Granddaughter	Moderate	Strongly agree	2
Tinyiko	44	F	Daughter	Good	Strongly agree	2
Vukona	37	F	Niece	Very good	Strongly agree	3
Doris	81	F	Wife	Moderate	Strongly agree	4
Violet	57	F	Wife	Very good	Neither agree nor disagree	1
Vangama	90	F	Sister	Good	Strongly agree	5
Dorothy	72	F	Wife	Good	Neither agree nor disagree	3
Hetisani	21	F	Granddaughter	Good	Strongly agree	2
Vutivi	61	F	Cousin-in-law	Good	Neither agree nor disagree	1
Fanisa	58	F	Daughter-in-law	Good	Neither agree nor disagree	2
Themba	37	M	Son	Very good	Strongly agree	0
Xisthembiso	69	F	Sister	Good	Strongly agree	2
Fatima	47	F	Paid caregiver	Very good	Somewhat agree	2
Enelo	25	F	Granddaughter	Good	Strongly agree	0
Nonisa	59	F	Daughter-in-law	Very good	Somewhat agree	2

^*^ Number of health conditions based on combined responses from the standard survey, the ethnographic survey and participant observation

### Caregiver Reported Health Conditions

Our interest in caregiver health, as discussed above, related to capacity to provide care, and to the possible extent to which underreporting of health problems might be influenced by other social considerations. As described in Table 3, most caregivers reported at least one chronic health condition in at least one of the methods. Overall, 44 conditions affecting 18 of the 21 caregivers were identified. The average number of health conditions per caregiver and the number of caregivers who reported one or more health problem differed between the standard survey, ethnographic observations, and ethnographic survey. Ethnographic observations overall identified the greatest number of health problems and the greatest number of caregivers with health problems, while the standard survey identified the least number of health problems and caregivers with health problems. The ethnographic survey identified eight health problems in 21 caregivers. In three cases, health conditions reported in the standard survey (one each of HIV, high cholesterol and kidney disease) were not identified or reported in either ethnographic observations or the ethnographic survey (Table 3). No method identified all health problems.

**Table 3:** Health problems reported by caregivers in response to different measures

	Standard survey	Ethnographic survey	Participant observation	Combined
Respondent count	21	13	21	21
HIV (1)	2	1	1	2
High blood pressure or hypertension (1)	5	5	6	6
Raised blood sugar or diabetes (1)				
Stroke (1)				
Angina/chest pain due to heart disease (1)				
Heart attack (1)				
High cholesterol (1)	1			1
Kidney disease (1)	1			1
Cancer (1)				
COPD or Asthma (1)				
Alzheimer’s disease or memory problems (1)			2	2
Parkinson’s disease (1)				
Hearing impairment (2)		1	1	1
Dental problems (2)		4	2	6
Vision impairment (2)		1	4	4
Eye pain		3	3	3
Musculo-skeletal pain		1	5	5
Mobility impairment		5	4	5
Hot flushes		2	1	2
Obesity or overweight			5	5
Dysmenorrhea		1		1
Fibroids			1	1
Gastroesophageal reflux or Heartburn		1	1	2

In the standard survey, respondents were explicitly asked if a healthcare worker had ever told them they had each condition marked (1) and then asked if they had any other health problems. In the ethnographic survey, respondents were asked to free-list any health conditions and then explicitly asked if they had problems affecting their eyes, ears or teeth (2). In participant observation conditions were elicited, spontaneously mentioned, noted or observed by the researcher

The difference in reporting is explained by the different data collection methods. The standard survey fieldworkers were relative strangers, compared to the ethnographers who developed a relationship with the participants through nine months of field work, and who learnt of different health conditions partly in relation to medicines the caregiver was taking. This suggests that the one report of HIV in the standard survey was a true case which the participant did not report to the ethnographic researchers, either because of familiarity or because they did not enquire directly. However, the case of reported kidney disease appears to be a data entry error: when asked explicitly (as follow up), the caregiver indicated that she had never had kidney disease. The participant who reported high cholesterol only in the standard survey regularly gave inconsistent reports, and told the ethnographers that he lied in the survey because he found the questions annoying. Of the 12 chronic health conditions specified in the standard survey, nine were not reported by any of the caregivers in the ethnographic research (Table 3).

When the ethnographic survey was conducted at the end of participant observation, 10 of the 14 caregivers reported 21 health problems (Table 3). Dental health problems or tooth loss, dysmenorrhea, heartburn, and HIV were not always reported in this survey. In contrast, longitudinal ethnographic observation identified overweight or obesity (5 cases), mobility impairment and related musculoskeletal pain (5), severe vision impairment due to cataract, comorbid with eye pain (3), dental problems (2), memory problems (2), uterine fibroids (1), hearing impairment (1) and heartburn (1) (Table 3). While HIV and hypertension were usually treated, other health problems including hot flushes and dental, vision and hearing problems were almost never treated.

### Caregiver Self-rated Health

Only three caregivers rated their health on the day of the standard survey as “moderate;” all others rated their health as either good (9) or very good (9). Seventeen caregivers agreed that they were healthy enough to care for the care recipient; the remaining four were equivocal, and ratings were not clearly linked to capacity to provide care. For example, two younger caregivers strongly agreed that they were healthy enough to care, despite self-rating their health as “moderate.” Conversely, one older caregiver indicated her health was good but neither agreed nor disagreed that she was healthy enough to care. Two caregivers rated their health as very good, but only “somewhat agreed” that they were healthy enough to provide care (Table 2).

Self-ratings of health did not clearly correlate with the caregivers’ combined health problems (Table 2). Of the three for whom no health problems were reported or observed, one rated their health “good” and the other two “very good.” The three caregivers who rated their health as moderate all had multiple health problems. Yet many others with multiple health problems rated their health as “good” or “very good.”

### Caregiver Health Case Studies

In the following case studies, we illustrate the diverse health experiences of caregivers. They draw attention to the complexity underlying responses to self-rated health questions on cross-sectional surveys.

#### Fatima

Fatima Chauke (47) was a non-family, paid caregiver for 82-year-old Katekani Mabaso. When we met her in August 2022, Fatima reported no mobility impairments and was observed to undertake or reported that she undertook heavy physical labor such as agricultural work and carting 25-L buckets of water and fruit. She rated her health as very good, despite appearing overweight, being treated for hypertension, and only agreeing “somewhat” that she was healthy enough to provide care. Fatima lived with Katekani and was on call to assist her. Everyday caregiving – cleaning the house, cooking meals, and assisting Katekani to bathe and dress – occupied her for only a fraction of the day. She reported doing agricultural and other outdoor work to keep busy, and to avoid being with Katekani when she became verbally abusive.

When we visited in February 2023, Fatima reported that she had “almost died” from a health problem that presented as a skin rash and sharp pains on the right-hand side of the body. She walked 1.5 km to the public health clinic to be assessed because she could not afford public transport. Nurses identified high blood pressure, and prescribed anti-hypertensive medicines supplementary to those Fatima already took. Fatima took the medicine, but when she did not feel better, she also visited a traditional healer, who charged ZAR1500 (by comparison her monthly caregiver salary was ZAR1000) but did not demand upfront payment. Fatima attributed her successful recovery to the traditional healer’s treatments.

While ill, Fatima was unable to care for Katekani, and Katekani’s sister came to the house daily to care for both women. Fatima recovered within a few weeks and continued caring for Katekani.

#### Doris

Doris Nkuna (81) was one of four caregivers who had serious vision impairments in one or both eyes due to untreated cataracts. We first met soon after she completed the standardized survey. She described her vision as cloudy. This limited her ability to care for her husband, who was at times physically and emotionally violent to her and other household members. Doris was unable to contribute to cooking, washing clothes or dishes, or cleaning the home, and relied on her daughter and granddaughter to do this and other caregiving labor that her poor vision (and general frailty) prevented her from doing. Yet Doris saw it as her duty to care for her husband, and self-identified as his primary caregiver. She worried about not being available to care for him, because her brother-in-law had once threatened to disinherit her for caring inadequately for him when she had not accompanied him to the clinic because of her own poor health.

Doris had been to hospital to have a cataract removed from one of her eyes. However, the surgery was unsuccessful and left her with chronic eye pain, which worsened on windy days. Soon after we met, her husband took her to a private eye doctor in the nearest town, who prescribed medicines and referred her for further cataract surgery. We accompanied Doris to the hospital on multiple occasions. During her first visit, she was assessed by a nurse, who told her she was eligible for free cataract surgery and that her name had been put on the waiting list. A month later, Doris (along with dozens of other patients whose names were on the waiting list) was called back for a second assessment by a visiting doctor, and her eligibility for surgery was confirmed. She had the cataract removed from one eye two months later, and was told she was eligible for surgery on her other eye six months later.

Shortly after returning home from cataract surgery, Doris experienced severe pain and impaired mobility from infected sores on her feet, and during a post-cataract surgery checkup, the nurse referred her for medical assessment. Doris was told that the foot sores were complications of the untreated hypertension condition. She began taking anti-hypertensive medicines, and her feet improved.

#### Vangama

Vangama (90) cared for her younger sister Xiluva (84) who, following a stroke, had been bedridden for almost ten years. Xiluva’s care needs were significant: she wore diapers; was unable to stand up or bathe herself; and often needed to be spoon-fed. Vangama, meanwhile, was being treated for hypertension and pain (possibly from arthritis), but the paracetamol tablets she received free from the public clinic did not help much. Vangama also had significant other impairments. She was completely deaf in one ear and had little hearing in the other. She had no sight in one eye due to a cataract and was losing vision in the other. She could walk, sometimes with the support of a stick, but this caused sharp hip pain, and she struggled to walk up and down the three steps at the front of her house. Vangama also had memory problems.

Although Vangama rated her health as very good and strongly agreed that she was healthy enough to care for Xiluva, we observed her health problems limiting her capacity to care. She was unable to lift Xiluva without assistance and so relied on others to help her bathe or move Xiluva. On several occasions we observed Xiluva making requests from bed (e.g. for food or water) that Vangama did not hear. Vangama needed to be reminded by her grandson, great-grandson, and Xiluva’s daughter to take her own and administer Xiluva’s medications. She insisted that she could see well enough to cook meals, but poor eyesight and failing memory meant that Vangama often lost things, including items used in caregiving routines such as soap. She often found them stashed in handbags or pockets.

Vangama had started caregiving fulltime after Kayise (Xiluva’s daughter) attempted to admit Xiluva to an aged care facility. Kayise had cared for Xiluva for more than nine years and could no longer cope. Vangama knew that no other family members were available to care for Xiluva, and she did not want her sister to be admitted to a facility. She cared for Xiluva until her death in February 2023. After Xiluva’s funeral, Vangama spoke more frequently of her hip pain, but said that it had not worsened. She explained that she had not referred to it earlier so to hide this from Xiluva.

#### Saseka

Saseka Mathe (29) rated herself as “moderately” healthy (the middle score) and having no health problems when she completed the standard survey in October 2022. She “strongly” agreed that she was healthy enough to care for her grandmother Namia (92), who had hypertension and mobility and vision impairments. Five months later, however, Saseka was seriously ill and told us she couldn’t remember how many trips to hospital she had made that year. We found out about these problems inadvertently, as we thought that her non-response to our requests to visit her were due to disinterest. But when we messaged her to say we would no longer visit unless she invited us, she asked us to keep visiting and explained that she had been too ill to answer or respond to our calls earlier in the year. She was in hospital recovering from the first of two surgeries that year, when she sent the message.

Saseka’s first surgery (in March) was to remove a lump from her throat (she never indicated the diagnosis); the second surgery (July) was to treat uterine fibroids. Throughout this period, she was tired and in pain much of the time. In addition, in May, she contracted mumps and moved into her mother’s house in a town 45 km away, leaving Namia to live alone and to be cared for by relatives who lived across the road. After each surgery, Saseka needed to rest so that her wounds could heal. She could not cook, clean, or even walk without pain, and her mother (Namia’s estranged daughter-in-law) moved into Namia’s homestead to care for Saseka, Saseka’s 9-year-old daughter, and Namia, despite continuing tension between the older women.

## Discussion

The three distinct means of enquiring about health reveal complexity and heterogeneity in what SRH meant to a small sample of caregivers in rural South Africa. Our findings, like others (Balaj & Eikemo, 2022; Mojola et al., 2022), reveal the lack of association between self-rated health and health conditions experienced by caregivers, while they highlight discrepancies in what people reported as health conditions according to when and how the data were collected (Mojola et al., 2022). The results suggest a need for carefully constructing health condition response categories informed by context specific evidence. They provide insights into the types of social factors which caregivers, of different ages, with different health conditions and in different caregiving arrangements, might consider when rating their own health and/or their ability to provide care.

The standard survey questions enquired about a constrained set of health worker diagnosed conditions. Three-quarters of these were not reported by any of the caregivers in the ethnographic study, in response to any of the three methods of enquiry. The caregivers were more commonly affected by and reported failing vision or hearing, or painful teeth for which they had little access to diagnosis or therapeutic services, and about which the standard survey did not enquire. This may partially explain the lack of association between self-rated health and health conditions (Balaj & Eikemo, 2022). But also, by failing to ask about functional conditions, we shaped the meanings of health for our respondents in the standard survey. These results indicate that quantitative surveys might be strengthened by (1) including lists of health conditions and/or symptoms that are informed by ethnographic work and/or context specific prevalence studies, and (2) by mentioning pathologies that are sometimes separated out (e.g. by healthcare systems, research instruments, or community norms) as “functional impairments” including dental, hearing, mobility and eye problems. Because caregivers are typically women, asking about common health conditions that only affect women, such as period pain and hot flushes, may also be important.

The negative impact of oral/eye/ear conditions as well as acute episodes of illness, both on caregiver wellbeing and ability to care, was apparent in the ethnographic research. Vangama could not hear her sister’s requests for assistance. Doris experienced eye pain and was unable to see to cook and clean, although these activities were core to caring for her husband. Saseka and Fatima, both relatively young and physically able, experienced rapid health declines following acute episodes of illness, which rendered them temporarily but entirely unable to provide physical care.

Our findings also draw attention to the value of supplementing data from cross sectional surveys that are not designed to measure the incidence of acute health problems (e.g. experiences by Fatima and Saseka) or undiagnosed conditions (e.g. Doris’ hypertension), with data generated through other methods. Our ethnographic data allowed us to track rapid changes in health status which could not be picked up in occasional surveys (e.g., annually or even less frequently). They could be used to inform the modification of standard survey questions, for example, the health conditions that respondents are asked explicitly about. Our findings also highlight the potential value of home visiting or community-led monitoring of at-risk homes or individuals, including those with substantial existing care needs (e.g. Vangama) and those that experience acute needs due to acute health problems (e.g. Saseka and Fatima). Such monitoring could be combined with clear reporting pathways and links to services to help those in need of healthcare or emergency support cover.

Self-rated health is a social measure, reflecting lived experience and socio-economic circumstances (Balaj & Eikemo, 2022; Dobreva & Posel, 2023; Jylhä, 2009; Mojola et al., 2022). Yet socio-material factors, such as past or current financial hardship or health behaviors, are relative to the individual’s culture, life course stage and gender (Balaj & Eikemo, 2022; Mojola et al., 2022). For example, some women in our study constructed positive health narratives to position themselves as capable caregivers. Their self-ratings of their health and ability to care appear to have been related more to their motivations to care, rather than their objective health status. Such motivations often include emotional attachments, cultural self-identity and culturally informed ideas of duty (Zarzycki et al., 2022). Caregiving was an important aspect of the gendered cultural identity of the caregivers in our study, as elsewhere on the continent (Schatz & Seeley, 2015).

When SRH is asked in a caregiving survey, its assessment will likely draw on the circumstances in which care is given. Both Vangama and Doris considered themselves healthy enough to provide care, despite being far from healthy (as might be assessed by a health worker) and not seeming to be well enough to provide optimal care. These women wanted to be healthy enough to care because it was expected of them, but also because no one else was available to take their place. Vangama’s positive health narrative was related both to her desire to prevent her sister being institutionalized, and the pleasure she derived from her sister’s company. She did not want her sister to be taken away, and thus she hid her pain and laughed off her hearing difficulties. Women assessed their health in relation to their social circumstances, specifically to prove their capacity to care for their households (see also Mojola et al., 2022).

While Vangama had a positive relationship with her sister, both Fatima and Doris reported abuse from their care recipients. These negative relations, rather than their objective health status, may have influenced their ambivalence that they were healthy enough to provide care. Fatima never questioned her ability to physically care for Katekani, except during her acute illness. Yet on several occasions she wondered how long she would be able to cope with Katekani’s provocations, and these likely contributed to her only somewhat agreeing that she was healthy enough to care, despite rating her health as very good. Doris consistently stated that she was unable to care for her husband because of her failing eyesight and frailty, but in the survey she responded uncertainly: she did not know if she was healthy enough to care. Yet she relied on her husband for property rights, and despite his violence, she was strongly attached to her status as a wife. She was expected (at least by her husband and his brother) to provide his care, despite her own poor health and frailty. She felt compelled to care, partly because of the threat that she would be disinherited (by her brother-in-law) of her husband’s property if she did not adequately do so. The expectations that influenced these women’s self-rating of health were highly gendered.

The self-assessments we document reflect the (non)availability of alternative care arrangements and indicate the importance of context in interpreting caregivers’ SRH – especially given the transitory and convenience-based nature of support for older persons in this community (Matina et al., 2025). These additional meanings have not been revealed by population wide studies (Balaj & Eikemo, 2022; Mojola et al., 2022), but may be important components of caregivers’ assessments of their health and ability to care.

We have illustrated the advantage of longitudinal ethnographic research in complementing and informing survey research. The resultant data offer important qualitative insights into possible meanings of caregivers’ self-ratings of health, which might in turn inform more appropriately constructed survey questions. Ethnographic research cannot uncover population-level patterns and trends, but ethnographic and other qualitative research can, and our findings indicate should, inform the design of surveys.

## Conclusion

What caregivers mean when they rate their health in particular ways varies depending on method. We found substantive discrepancies across methods, including in key health conditions which impacted people’s ability to provide care. The results indicate that caregivers’ self-ratings of their health reflect social circumstances, and that these circumstances influence how caregivers rate their health and report their capacity to care. The nature of the relationship of caregiver and care recipient, gendered expectations about who should provide care (despite health status), and the availability and cultural acceptability of alternative care arrangements, all influence preparedness to care, despite the health status of the care provider. We have highlighted the importance of triangulating data generated from different methods, to gain a more comprehensive picture of caregivers’ health, and so provided a compelling case for mixed-methods studies to capture changes in health status at scale and over time. Such studies are likely to provide deep insight into the meanings which caregivers attribute to health ratings, changes in health status attributable to caregiving, and how such changes impact on care recipients. They also provide a model for community -based monitoring that could improve support provided to caregivers, for example during acute illness.

## Data Availability

No datasets were generated or analysed during the current study.

## Declarations

### Disclaimer

The funders had no role in study design, data collection and analysis, decision to publish, or preparation of the manuscript.

## References

[CR1] Andresen, E. M., Malmgren, J. A., Carter, W. B., & Patrick, D. L. (1994). Screening for depression in well older adults: Evaluation of a short form of the CES-D (Center for Epidemiologic Studies Depression Scale). *American Journal of Preventive Medicine,**10*(2), 77–84. 10.1016/S0749-3797(18)30622-6 8037935

[CR2] Au, N., & Lorgelly, P. K. (2014). Anchoring vignettes for health comparisons: An analysis of response consistency. *Quality Of Life Research,**23*(6), 1721–1731. 10.1007/s11136-013-0615-2 24384738 10.1007/s11136-013-0615-2

[CR3] Balaj, M., & Eikemo, T. A. (2022). Sick of social status: A bourdieusian perspective on morbidity and health inequalities. *Sociology Of Health & Illness,**44*(8), 1214–1250. 35779001 10.1111/1467-9566.13512 PMC9540620

[CR4] Bassil, D. T., Farrell, M. T., Weerman, A., Guo, M., Wagner, R. G., Brickman, A. M., Glymour, M. M., Langa, K. M., Manly, J. J., Tipping, B., Butler, I., Tollman, S., & Berkman, L. F. (2023). Feasibility of an online consensus approach for the diagnosis of cognitive impairment and dementia in rural South Africa. *Alzheimer’s & Dementia: Diagnosis, Assessment & Disease Monitoring,**15*(2), Article e12420. 10.1002/dad2.12420 10.1002/dad2.12420 PMC10072202 37025188

[CR5] Bassil, D. T., Farrell, T. F., Wagner, R. G., Brickman, A. M., Glymour, M. M., Langa, K. M., Manly, J. J., Salinas, J., Tipping, B., Tollman, S., & Berkman, L. F. (2022). Cohort profile update: Cognition and dementia in the Health and Aging in Africa Longitudinal Study of an INDEPTH community in South Africa (HAALSI dementia). *International Journal of Epidemiology,**51*(4), e217–e226. 10.1093/ije/dyab250 34871405 10.1093/ije/dyab250 PMC9365629

[CR6] Bom, J., Bakx, P., Schut, F., & Van Doorslaer, E. (2019). The impact of informal caregiving for older adults on the health of various types of caregivers: A systematic review. *The Gerontologist,**59*(5), e629–e642. 10.1093/geront/gny137 30395200 10.1093/geront/gny137 PMC6850889

[CR7] Calvey, B., Power, J. M., Maguire, R., de Andrade Moral, R., & de Lara, I. A. R. (2024). Do discrepancies between subjective and objective health shift overtime in later life? A markov transition model. *Social Science & Medicine,**362*, Article 117441. 10.1016/j.socscimed.2024117441 39488954 10.1016/j.socscimed.2024.117441

[CR8] Dobreva, R., & Posel, D. (2023). “Your Health at Present”: Are Patterns of Reporting Heterogeneity in Self-rated Health Gendered? *Applied Research In Quality Of Life,**18*(5), 2197–2226. 10.1007/s11482-023-10183-y

[CR9] Ginsburg, C., Collinson, M. A., Gómez-Olivé, F. X., Gross, M., Harawa, S., Lurie, M. N., Mukondwa, K., Pheiffer, C. F., Tollman, S., Wang, R., & White, M. J. (2021). Internal migration and health in South Africa: Determinants of healthcare utilisation in a young adult cohort. *Bmc Public Health,**21*, 554. 10.1186/s12889-021-10590-6 33743663 10.1186/s12889-021-10590-6 PMC7981972

[CR10] Gómez-Olivé, F. X., Montana, L., Wagner, R. G., Kabudula, C. W., Rohr, J. K., Kahn, K., Bärnighausen, T., Collinson, M., Canning, D., Gaziano, T., Salomon, J. A., Payne, C. F., Wade, A., Tollman, S. M., & Berkman, L. (2018). Cohort profile: Health and ageing in Africa: A longitudinal study of an INDEPTH community in South Africa (HAALSI). *International Journal of Epidemiology,**47*(3), 689–690j. 10.1093/ije/dyx247 29325152 10.1093/ije/dyx247 PMC6005147

[CR11] Guillemin, M., & Gillam, L. (2004). Ethics, reflexivity, and “ethically important moments” in research. *Qualitative Inquiry,**10*(2), 261–280. 10.1177/1077800403262360

[CR12] Ice, E., Mojola, S. A., Angotti, N., Gómez-Olivé, F. X., & Houle, B. (2022). Making sense of troubled livelihoods: Gendered expectations and poor health narratives in rural South Africa. *Gender & Society,**36*(5), 735–763. 10.1177/08912432221114877

[CR13] Jylhä, M. (2009). What is self-rated health and why does it predict mortality? Towards a unified conceptual model. *Social Science & Medicine,**69*(3), 307–316. 10.1016/j.socscimed.2009.05.013 19520474 10.1016/j.socscimed.2009.05.013

[CR14] Kahn, K., Collinson, M. A., Gómez-Olivé, F. X., Mokoena, O., Twine, R., Mee, P., Afolabi, S. A., Clark, B. D., Kabudula, C. W., Khosa, A., & Khoza, S. (2012). Profile: Agincourt health and socio-demographic surveillance system. *International Journal of Epidemiology,**41*(4), 988–1001. 10.1093/ije/dys115 22933647 10.1093/ije/dys115 PMC3429877

[CR15] Letelioer, A., Madero-Cabib, I., Undurraga, E. A., & Pérez-Cruz, P. (2021). Lifetime socioeconomic determinants of health trajectories among older adults. *Advances in Life Course Research,**49*, Article 100415. 10.1016/j.alcr.2021.100415 34733129 10.1016/j.alcr.2021.100415 PMC8562571

[CR16] Lewis, C. T., Jang, Y., Elayoubi, J., Sanchez, V. A., Arnold, M. L., Toman, J., & Haley, W. E. (2025). Racial differences subjective rating hearing impairment. *The Gerontologist,**65*(5), gnaf029. 10.1093/geront/gnaf029 39878698 10.1093/geront/gnaf029

[CR17] Li, W., Xu, Z., & Tang, W. (2024). Gender differences in self-rated health among older adults in the Chinese workforce. *Frontiers in Public Health*. 10.3389/fpubh.2024.1450045 10.3389/fpubh.2024.1450045 PMC11659123 39712317

[CR18] Manderson, L., Brear, M. R. , Rusere, F., Farrell, M., Gómez-Olivé, F. X., Berkman, L., Kahn, K., & Harling, G. (2022). Protocol: The complexity of informal caregiving for Alzheimer's disease and related dementias in rural South Africa. *Wellcome Open Research**7*(220), 220. 10.12688/wellcomeopenres.18078.1 10.12688/wellcomeopenres.18078.1 PMC10394391 37538406

[CR19] Matina, S. S., Manderson, L., Brear, M., Rusere, F., Gómez-Olivé, F. X., Kahn, K., & Harling, G. (2025). Distribution of informal caregiving for older adults living with or at risk of cognitive decline within and beyond family in rural South Africa. *The Journals of Gerontology, Series B: Psychological Sciences and Social Sciences,**80*(5), 008. 10.1093/geronb/gbaf008 10.1093/geronb/gbaf008 PMC11974393 39862221

[CR20] Medvedyuk, S., & Raphael, D. (2023). Defining health through a critical materialist political economy lens. *Global Health Promotion*. 10.1177/17579759231194600 10.1177/17579759231194600 PMC11363467 37823385

[CR21] Mojola, S. A., Ice, E., Schatz, E., Angotti, N., Houle, B., & Gómez-Olivé, F. X. (2022). The meaning of health in rural South Africa: Gender, the life course, and the socioepidemiological context. *Population and Development Review,**48*(4), 1061–1095. 10.1111/padr.12494

[CR22] Mokgatle, M., & Madiba, S. (2023). Community perceptions of HIV stigma, discriminatory attitudes, and disclosure concerns: A health facility-based study in selected health districts of South Africa. *International Journal of Environmental Research and Public Health,**20*(14), 6389. 10.3390/ijerph20146389 37510621 10.3390/ijerph20146389 PMC10379360

[CR23] Morse, J. M. (2015). Critical analysis of strategies for determining rigor in qualitative inquiry. *Qualitative Health Research,**25*(9), 1212–1222. 10.1177/1049732315588501 26184336 10.1177/1049732315588501

[CR24] Onwuegbuzie, A., & Combs, J. (2010). Emergent data analysis techniques in mixed methods research: A synthesis. In A. Tashakkori & C. Teddlie (Eds.), *Sage Handbook of Mixed Methods in Social and Behavioral Research* (2nd ed., pp. 397–430). Sage.

[CR25] Payne, C. F., Mall, S., Kobayashi, L., Kahn, K., & Berkman, L. (2020). Life-course trauma and later life mental, physical, and cognitive health in a postapartheid South African population: Findings from the HAALSI study. *Journal of Aging and Health,**32*(9), 1244–1257. 10.1177/0898264320913450 32207348 10.1177/0898264320913450 PMC8054553

[CR26] Ralston, M., Schatz, E., Menken, J., Gómez-Olivé, F. X., & Tollman, S. (2015). Who benefits – or does not – from South Africa’s old age pension? Evidence from characteristics of rural pensioners and non-pensioners. *International Journal of Environmental Research and Public Health,**13*(1), Article 85. 10.3390/ijerph13010085 26712777 10.3390/ijerph13010085 PMC4730476

[CR27] Schatz, E., & Seeley, J. (2015). Gender, ageing and carework in East and Southern Africa: A review. *Global Public Health,**10*(10), 1185–1200. 10.1080/17441692.2015.1035664 25947225 10.1080/17441692.2015.1035664 PMC4888771

[CR28] van Ginneken, J. & Groenewold, W. (2012). A single- vs. multi-item self-rated health status measure: a 21-country study. *The Open Public Health Journal, *5:1–9. http://benthamopen.com/contents/pdf/TOPHJ/TOPHJ-5-1.pdf.

[CR29] World Health Organization [WHO]. (2018). *Global Reference List of 100 Core Health Indicators*. Retrieved from https://score.tools.who.int/fileadmin/uploads/score/Documents/Enable_data_use_for_policy_and_action/100_Core_Health_Indicators_2018.pdf.

[CR30] Xiao, Z. W., Lee, J., & Wu, A. L. (2024). Social determinants of self-rated health: Patient-provider communication, social support, socioeconomics, and demographics among different ethnic groups in the U.S. *Howard Journal of Communications,**35*(1), 100–117. 10.1080/10646175.2023.2256772

[CR31] Yu, S. T., Houle, B., Manderson, L., Jennings, E. A., Tollman, S. M., Berkman, L. F., & Harling, G. (2022). The double-edged role of accessed status on health and well-being among middle- and older-age adults in rural South Africa: The HAALSI study. *SSM - Population Health,**19*, Article 101154. 10.1016/j.ssmph.2022.101154 35855969 10.1016/j.ssmph.2022.101154 PMC9287360

[CR32] Yu, X., Kabudula, C. W., Wagner, R. G., Bassil, D. T., Farrell, M. T., Tollman, S. M., Kahn, K., Berkman, L. F., Rosenberg, M. S., & Kobayashi, L. C. (2024). Mid-life employment trajectories and subsequent memory function and rate of decline in rural South Africa, 2000–22. *International Journal of Epidemiology,**53*(2), Article dyae022. 10.1093/ije/dyae022 38365967 10.1093/ije/dyae022 PMC10873492

[CR33] Zarzycki, M., Seddon, D., Bei, E., Dekel, R., & Morrison, V. (2022). How culture shapes informal caregiver motivations: A meta-ethnographic review. *Qualitative Health Research,**32*(10), 1574–1589. 10.1177/10497323221110356 35737473 10.1177/10497323221110356 PMC9411702

